# Multimodal Large Language Model-Enabled Machine Intelligent Fault Diagnosis Method with Non-Contact Dynamic Vision Data

**DOI:** 10.3390/s25185898

**Published:** 2025-09-20

**Authors:** Zihan Lu, Cuiying Sun, Xiang Li

**Affiliations:** 1 Key Laboratory of Education Ministry for Modern Design and Rotor-Bearing System, Xi’an Jiaotong University, Xi’an 710049, China; 2 State Key Laboratory of Engine and Powertrain System, Weichai Power Co., Ltd., Weifang 261061, China; suncuiy@weichai.com

**Keywords:** event camera, fault diagnosis, multimodal large models, dynamic vision

## Abstract

Smart manufacturing demands ever-increasing equipment reliability and continuous availability. Traditional fault diagnosis relies on attached sensors and complex wiring to collect vibration signals. This approach suffers from poor environmental adaptability, difficult maintenance, and cumbersome preprocessing. This study pioneers the use of high-temporal-resolution dynamic visual information captured by an event camera to fine-tune a multimodal large model for the first time. Leveraging non-contact acquisition with an event camera, sparse pulse events are converted into event frames through time surface processing. These frames are then reconstructed into a high-temporal-resolution video using spatiotemporal denoising and region of interest definition. The study introduces the multimodal model Qwen2.5-VL-7B and employs two distinct LoRA fine-tuning strategies for bearing fault classification. Strategy A utilizes OpenCV to extract key video frames for lightweight parameter injection. In contrast, Strategy B calls the model’s built-in video processing pipeline to fully leverage rich temporal information and capture dynamic details of the bearing’s operation. Classification experiments were conducted under three operating conditions and four rotational speeds. Strategy A and Strategy B achieved classification accuracies of 0.9247 and 0.9540, respectively, successfully establishing a novel fault diagnosis paradigm that progresses from non-contact sensing to end-to-end intelligent analysis.

## 1. Introduction

For a long time, fault diagnosis methods based on vibration signal analysis have dominated the field of industrial equipment condition monitoring. This technical paradigm typically follows a standardized workflow: sensor deployment → signal acquisition → feature engineering → model classification [1]. In the traditional diagnostic process, the core technical challenge is concentrated on how to extract features from one-dimensional time-domain vibration signals that can effectively distinguish between different fault modes. These analysis methods are primarily categorized as follows.

Time-domain analysis directly calculates various statistical indicators of the vibration signal, such as RMS, peak, kurtosis, and crest factor, to measure the signal’s energy and impact characteristics [2]. The advantage of this approach lies in its simplicity and intuitive physical meaning, but its sensitivity to incipient and weak faults is relatively low. Frequency-domain analysis transforms the time-domain signal into the frequency domain for analysis via the fast Fourier transform. Under steady-state operating conditions, different mechanical faults produce specific characteristic frequency peaks. FFT analysis is easy to implement and highly effective, but its main limitation is insufficient frequency resolution for non-stationary or short-transient signals. Time-frequency analysis aims to integrate information from both the time and frequency dimensions. This makes it particularly suitable for analyzing signals under non-stationary conditions, such as variable speeds and loads. Common techniques include the short-time Fourier transform, Wigner–Ville distribution, and the wavelet transform. Among these, the wavelet transform is particularly favored for its multiresolution capability to capture transient impact components in signals.

Following feature extraction, researchers typically employ various classifiers to determine the health status of the equipment [3].

Traditional machine learning models such as support vector machines, k-nearest neighbors, and decision trees are widely used. However, the performance of these methods is highly dependent on the quality of manually engineered features. In contrast, deep learning models, represented by convolutional neural networks, have enabled end-to-end diagnosis. This approach can directly take one-dimensional vibration signals as input to automatically learn and extract fault features. This not only eliminates the tedious process of feature design but also generally achieves higher diagnostic accuracy [4].

An event camera is a novel bio-inspired vision sensor. Each of its pixels operates independently and asynchronously. The camera does not capture images at a fixed rate. Instead, it only outputs the precise time, location, and polarity of a brightness change when one is perceived. This process forms a data stream. Here are the advantages of dynamic vision compared to traditional vibration signals [5]:

(1) Sensing Paradigm and Deployment: Traditional methods require sensors to be in physical contact with the equipment under test. This makes the installation process cumbersome, maintenance costly, and deployment in difficult areas extremely challenging. It may even interfere with the system’s own dynamic characteristics due to the mass-loading effect. In stark contrast, dynamic vision technology, represented by event cameras, offers a completely non-contact solution. It enables remote sensing from a safe distance, greatly simplifying deployment and ensuring non-intrusive measurements [6].
(2) Data Fidelity and Information Dimension: Traditional accelerometers are limited by their sampling frequency. This often makes them inadequate for capturing the high-frequency transient signals caused by incipient faults. This can easily lead to signal aliasing or information loss. Dynamic vision, especially event cameras, offers microsecond-level temporal resolution. Their event-driven mechanism accurately captures these high-speed transient processes. They also naturally filter out static background noise, allowing them to acquire key fault features with extremely low data redundancy [7].
(3) Environmental Adaptability and Interference Immunity: Strong electromagnetic environments in industrial sites pose a severe challenge for traditional electronic sensors. Electromagnetic interference (EMI) often overwhelms weak fault signals. As an optical measurement technique, dynamic vision is inherently immune to such interference. Furthermore, its high dynamic range (HDR) allows it to operate stably under extreme or changing lighting conditions. This demonstrates excellent environmental robustness and ensures signal reliability [8].
(4) Monitoring Scope and Diagnostic Efficiency: Traditional methods are essentially based on point sensing, where each sensor provides information from only a single location. To obtain the complete vibration pattern of a device, an expensive and complex sensor array must be deployed. In contrast, a single dynamic vision sensor can cover a wide field of view. This enables synchronous, full-field monitoring of multiple components or even an entire surface. This leap from point-to-surface monitoring significantly improves efficiency. It also provides rich spatial information for comprehensive diagnostics like operational deflection shape analysis [9].

Concurrently, multimodal large models, such as the Qwen2.5-VL series, can process multiple information streams. These include text, images, and even video. Their powerful capabilities in visual grounding and chart parsing offer new approaches for analyzing dynamic vision data. These models can be used to jointly analyze the micro-motion video streams captured by event cameras, achieving an organic integration of them.

Preliminary validation shows that this framework significantly improves diagnostic accuracy over traditional methods. This has been tested on standard bearing fault datasets and in real industrial field tests. Ultimately, this solution provides a novel diagnostic capability that is non-contact, end-to-end, and high-precision. It opens a new, generalizable path for intelligent industrial systems [10].

## 2. Materials and Methods

### 2.1. From Event Camera Raw Data to Training-Ready Video

**Figure 1:**
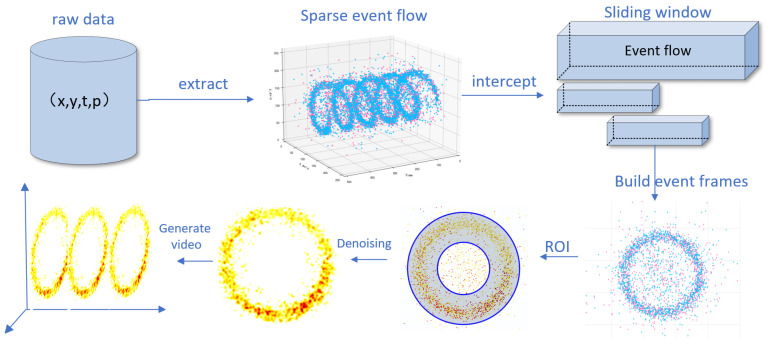
Workflow for event camera raw data to video generation.

In this study, we utilize raw data from an event camera, collected during motor operation, for fault diagnosis. Unlike conventional frame-based cameras, an event camera operates on an asynchronous pixel response mechanism. Each pixel independently and continuously monitors for logarithmic changes in its received light intensity. When the change in log intensity at a pixel location (x,y) exceeds a predefined threshold, that pixel immediately generates an event, which is recorded as a four-tuple: (x,y,t,p) [11]. In this tuple, x and y are the pixel coordinates, t is the precise timestamp (with microsecond-level resolution), and p is the polarity (+1 indicates an increase in brightness, while −1 indicates a decrease). However, this unstructured data stream is fundamentally incompatible with the structured input format required for fine-tuning. Furthermore, the raw event data inevitably contains noise induced by thermal effects or circuit crosstalk, which interferes with the extraction of meaningful signals [12]. To address these challenges, this paper proposes a complete pipeline for data preprocessing and dataset construction, with the overall process illustrated in Figure 1.

#### 2.1.1. Time Surface Representation

To transform the preprocessed, sparse event stream into a dense, frame-based representation suitable for convolutional neural networks, we adopt the time surface (SAE) construction method. As a classic representation for event streams, the SAE encodes the spatiotemporal information of a sequence of discrete events onto a 2D plane. This creates a feature map rich in dynamic information, effectively bridging the gap between asynchronous event data and synchronous neural network models.

Specifically, to fully preserve the critical information regarding the direction of brightness change (i.e., event polarity), we construct independent SAEs for positive (p = +1, brightness increase) and negative (p = −1, brightness decrease) events. This approach results in a more informative dual-channel feature representation [13].

The conversion process is performed independently within each fixed-duration time window Δt. The core steps and their mathematical formulations are detailed below:

(1) Timestamp Normalization
  To eliminate the influence of absolute timestamps and to focus on the relative recency of events within the current time window, we first linearly normalize the timestamps $t_{j}$ of all events within the window to the interval [0,1]:
  (1) $${\hat{t}}_{j}=\frac{t_{j}-t_{\min}}{t_{\max}-t_{\min}}$$
  where $t_{j}$ is the original timestamp of event $e_{j}$, while $t_{\min}$ and $t_{\max}$ are the start and end times of the current time window Δt, respectively. The variable ${\hat{t}}_{j}$ represents the normalized timestamp.
(2) Exponential Decay Kernel
  The contribution of each event to the time surface is defined by an exponential decay kernel function. This function assigns a higher weight to more recent events, thereby creating a “brighter” trace on the surface. Its mathematical expression is:
  (2) $${\mathrm{value}}_{j}=\exp\left(-\tau \;\cdot \;\left(1-{\hat{t}}_{j}\right)\right)$$
  where τ is a predefined time decay constant that controls the “memory” duration of the event history.
(3) Surface Update with Polarity Separation
  Based on the polarity $p_{j}$ of each event, the corresponding SAE is updated at the pixel location $\left(x_{j},y_{j}\right)$ by taking the historical maximum value. This operation ensures that at the same pixel location, only the event with the highest weight (i.e., the most recent one) can refresh the surface value, thus effectively allowing the surface to record the “trace” of the last activation at each pixel. The update rule is as follows [14]:
  (3) $$S_{p}\left(x_{j},y_{j}\right)=\max\left(S_{p,\mathrm{old}}\left(x_{j},y_{j}\right),{\mathrm{value}}_{j}\right)\;\mathrm{where}\;p\in \left\{+1,-1\right\}$$
  where $S_{p}$ represents the time surface for polarity *p* ($S_{+1}$ is $S_{\mathrm{pos}}$, and $S_{-1}$ is $S_{\mathrm{neg}}$), and $S_{p,\mathrm{old}}$ is the value of that pixel before the update.
(4) Final Surface Normalization
  After iterating through all events within a time window, we perform a min–max normalization on the generated positive and negative time surfaces separately. This operation enhances the contrast of the SAE frames and standardizes their value range for subsequent processing by linearly scaling the pixel values of each surface to the interval [0,1]. For any given time surface $S_{p}$, the normalized surface $S_{p}^{\prime }$ is calculated as follows [15]:
  (4) $$S_{p}^{\prime }=\frac{S_{p}-\min\left(S_{p}\right)}{\max\left(S_{p}\right)-\min\left(S_{p}\right)}$$
  This step is executed only if the surface contains valid events (i.e., $\max(S_{p})>0$).

Through this complete four-step process, the sparse event stream within each time window Δt is successfully converted into a pair of standardized SAE frames, $S_{\mathrm{pos}}^{\prime }$ and $S_{\mathrm{neg}}^{\prime }$, which are rich in spatiotemporal dynamics.

#### 2.1.2. Spatial Density-Based Denoising

Event camera sensors are inherently sensitive to factors like thermal effects and circuit crosstalk. This leads to the unavoidable presence of noise in the output event stream. This noise typically manifests as spatiotemporally isolated and uncorrelated “salt-and-pepper” events. If not addressed, these spurious events can introduce erroneous artifacts during the construction of the time surface. This severely interferes with the extraction of effective features and ultimately compromises the accuracy and robustness of the fault diagnosis model.

To effectively suppress such noise, we employ a filtering algorithm based on spatial neighborhood density. The core idea of this method stems from a fundamental assumption: valid events generated by real-world scene changes (such as bearing vibrations) exhibit local spatiotemporal correlation, whereas noise events tend to exist in isolation. Therefore, by evaluating the neighborhood support for each event, it is possible to effectively distinguish it from noise.

Specifically, this denoising process is executed independently within each small time slice (Δt) immediately prior to the generation of a time surface frame. This strategy of applying spatial filtering within temporal slices leverages the spatial clustering properties of events while preserving the inherent high temporal resolution of the event stream. Our implementation adopts an efficient two-step approach: first, a 2D histogram of event counts (count_map) is created for all events within the current time slice; then, each event in the slice is iterated through again, and its neighborhood density is rapidly calculated by querying the count_map. For any given event $e_{i}=\left(x_{i},y_{i},t_{i},p_{i}\right)$ within the time slice, we calculate the total number of events $D(e_{i})$, within its spatial neighborhood $N(e_{i})$ of radius *r*. This process can be expressed by the following equation [16]:

(5) $$D\left(e_{i}\right)=\sum_{e_{j}\in \Delta t,{∥\left(x_{j},y_{j}\right)-\left(x_{i},y_{i}\right)∥}_{\infty }\leq r}1$$

where ${∥\;\cdot \;∥}_{\infty }$ denotes the Chebyshev distance, which defines a square neighborhood centered on event $e_{i}$. In this study, we set the neighborhood radius r=1, corresponding to a 3×3 pixel window. If the total number of events in an event’s neighborhood, $D(e_{i})$, is less than a predefined minimum support threshold $N_{\min}$, the event is classified as an isolated noise point and is discarded. Based on our code implementation, we set $N_{\min}=2$. This choice of threshold implies that an event must have at least one accompanying neighbor within the same 3×3 spatiotemporal slice to be considered a valid signal. This setting achieves a balance between noise removal and signal fidelity, as it effectively filters isolated noise points while being able to preserve the endpoints of motion trajectories. The filtering condition is:

(6) $$e_{i}\;\mathrm{is}\;\mathrm{noise}\;\mathrm{if}\;D\left(e_{i}\right)<N_{\min}$$

**Figure 2:**
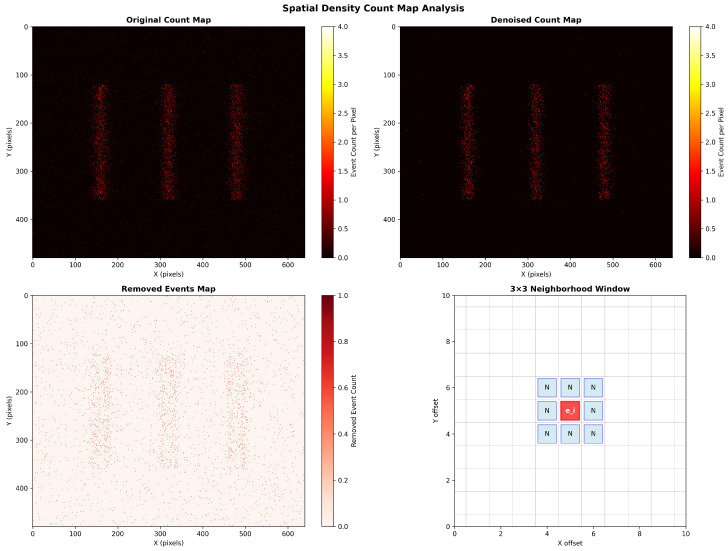
Original, denoised, and noise images obtained from simulated event camera data.

This lightweight spatial filtering method effectively suppresses noise while incurring minimal computational overhead. Furthermore, it maximally preserves the original spatiotemporal structure of the signal, laying a solid foundation for the subsequent construction of high-quality time surface representations. The denoising effect using simulated data is illustrated in Figure 2.

#### 2.1.3. ROI Cropping

The raw field of view from the event camera is large. It contains significant background areas irrelevant to the bearing’s status, as well as interference events caused by ambient light changes. To reduce the computational load of subsequent processing and enable the model to focus on the signal source carrying critical fault features, we designed and implemented a hierarchical, two-stage region of interest (ROI) cropping strategy [17].

(1) Coarse ROI Determination via Event Density Statistics
  The objective of this stage is to automatically localize the macroscopic region containing the key bearing structures in a data-driven manner. This process operates directly on the entire raw event stream over a period of time, with its core based on the 1D projection analysis of event density [18]. First, we construct the event density histograms, $H_{x}$ and $H_{y}$, by projecting all events onto the x- and y-axes, respectively:
  (7) $$H_{x}\left(j\right)=\sum_{i=1}^{N}\delta \left(x_{i}-j\right),\;H_{y}\left(k\right)=\sum_{i=1}^{N}\delta \left(y_{i}-k\right)$$
  where δ(·) is the Kronecker delta function and *N* is the total number of events.
  Subsequently, on the x-axis density histogram, we perform a convolution with a 3-point uniform averaging kernel, K=[1/3,1/3,1/3], to obtain a smoothed density curve, $S_{x}\left(j\right)=\left(H_{x}*K\right)\left(j\right)$. A multi-constraint peak detection algorithm is then applied to robustly identify the peak locations *P* corresponding to the three core vertical lines of the bearing:
  (8) $$P=\{j:S_{x}\left(j\right)\geq \eta_{h}\wedge d\left(j,P\setminus \left\{j\right\}\right)\geq \delta_{\min}\wedge \mathrm{prom}\left(j\right)\geq \eta_{p}\}$$
  where $\eta_{h}$, $\delta_{\min}$, and $\eta_{p}$ are the thresholds for height, distance, and prominence, respectively.
  Concurrently, the vertical boundaries of the effective event area, $y_{\min}$ and $y_{\max}$, are determined from the y-axis density histogram using an adaptive threshold $\tau_{y}=0.1\;\cdot \;\max\left(H_{y}\right)$. A similar thresholding strategy is applied locally for each detected x-axis peak to determine its precise left and right boundaries. Through this automated analysis, we can ascertain the optimal ROI parameters for data under various operating conditions, which are then used for the initial coarse filtering of the raw sparse event stream.
(2) Content-based Dynamic Fine-Tuning
  Following the Stage 1 filtering and the subsequent generation of time surface (SAE) frames, we execute a second stage of dynamic cropping to ensure the final output video frames are as compact as possible. This stage operates on the generated SAE frame sequence, T(x,y). We obtain an aggregated activity map, M(x,y), by projecting the SAE sequence along the time axis:
  (9) $$M\left(x,y\right)=⊮\left[\bar{T}\left(x,y\right)>\tau_{d}\right],\;\mathrm{where}\;\bar{T}\left(x,y\right)=\max_{t}T\left(x,y,t\right)$$
  where ⊮[·] is the indicator function and $\tau_{d}$ is an activity threshold. Then, based on this aggregated map, a minimal bounding box $\left(x_{\min}^{d},y_{\min}^{d},x_{\max}^{d},y_{\max}^{d}\right)$ that encloses all non-zero pixels is calculated, with an additional small padding parameter ρ. Finally, every frame in the sequence is uniformly and precisely cropped according to this bounding box and resized to the final target dimensions.

**Figure 3:**
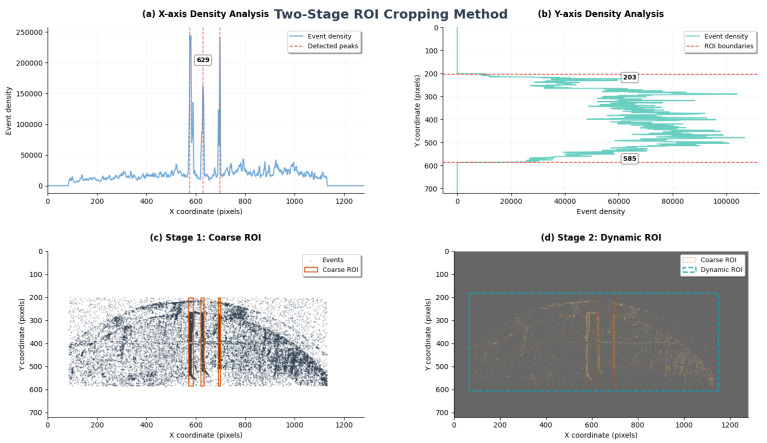
Two-stage ROI cropping method: coarse ROI filtering and target region.

This two-stage strategy, which combines statistical coarse detection with content-adaptive fine-tuning, ensures that the visual features extracted from the raw data have the highest possible signal-to-noise ratio and information density, laying a solid foundation for high-precision fault diagnosis [19]. This strategy balances data-driven robustness with content-adaptive precision, and its process is illustrated in Figure 3.

### 2.2. Construction of the Fine-Tuning Dataset

#### 2.2.1. Dataset Format Specification

To construct a fault diagnosis dataset that can be effectively understood and processed by large multimodal models, we formalize each diagnostic task as an independent sample. The entire diagnostic analysis dataset, $X_{\mathrm{inference}}$, can be represented as a collection of samples: $X_{\mathrm{inference}}=\left\{x_{1},x_{2},\ldots ,x_{n}\right\}$  [20].

As per our code implementation, each diagnostic sample $x_{i}$ is designed as a multimodal data unit containing four key fields. Its structure is defined as follows:

(10) $$x_{i}=\left\{{\mathrm{instruction}}_{i},{\mathrm{input}}_{i},{\mathrm{output}}_{i},{\mathrm{videos}}_{i}\right\}$$

This structure closely integrates natural language instructions with visual video data. It forms a standard multimodal learning paradigm suitable for complex reasoning tasks. These tasks require a joint understanding of both text and video content. The data type and content definition for each field in this study are detailed in Table 1.

#### 2.2.2. Synonymous Instruction Augmentation

A model can easily overfit if it is exposed only to a single, fixed instruction text during the training phase. This leads to poor performance when faced with the diverse range of queries encountered in the real world.

To overcome this limitation, we introduce a data augmentation strategy termed synonymous instruction augmentation. Its core objective is to programmatically generate instructions for each diagnostic sample. These instructions are semantically consistent yet diverse in their syntactic structure and lexical choices [21,22].

#### 2.2.3. Template-Based Parametric Instruction Generation

Our augmentation method is based on a pre-constructed library of instruction templates. Let this template library be $T=\{T_{1},T_{2},\ldots ,T_{M}\}$, which contains M=100 synonymous sentence templates with placeholders. For any given sample $x_{i}$ in the dataset, its associated dynamic parameter (in this study, the motor’s rotational speed) is denoted as $p_{i}$ [23,24].

The final instruction, instruction_i_, for sample $x_{i}$ is generated through the following two-step process:

(1) Random Template Sampling: A template $T_{k}$ is randomly and uniformly sampled from the library *T*.
  (11) $$T_{k}∼\mathrm{Uniform}\left(T\right)$$
(2) Parametric Instantiation: The specific parameter value $p_{i}$ of sample $x_{i}$ is inserted into the placeholder(s) of the sampled template $T_{k}$ to generate the final instruction text.
  (12) $${\mathrm{instruction}}_{i}=\mathrm{format}\left(T_{k},p_{i}\right)$$

For instance, for an outer-ring fault video with a rotational speed of ‘5 Hz‘, the instruction could be any of the following forms:

- “At 5 Hz, use the video to determine if the bearing is in the innerH, outerH, or normal state.”
- “With the video at 5 Hz rotation, classify the bearing as innerH, outerH, or normal.”
- “From this video at 5 Hz, please indicate: is the bearing innerH, outerH, or normal?”
- “Examine the video at 5 Hz and specify: innerH, outerH, or normal.”

#### 2.2.4. Enhancing Model Generalization Through Instruction Augmentation

By implementing synonymous instruction augmentation, we significantly enhance the richness and diversity of the dataset’s textual modality. This strategy compels the model to learn the core diagnostic intent from different linguistic expressions, rather than merely memorizing specific word orders or sentence structures. This effectively improves the model’s generalization ability on unseen instructions. It also lays a solid foundation for building more intelligent and interactively natural fault diagnosis systems [25].

### 2.3. Lightweight Video Fine-Tuning of Qwen2.5-VL

To adapt the pre-trained Qwen2.5-VL model for the video-based fault diagnosis task, this study employs a parameter-efficient fine-tuning (PEFT) framework. The objective is to transfer the model’s capabilities to the target domain with minimal training cost.

(1) Multimodal Feature Encoding: We encode the features from different modalities.
  - For video spatiotemporal features, the video sequence is processed by a Conv3D module to capture local features. It is then fed into a hierarchical vision transformer encoder, composed of *M* window attention modules and one full attention module, to extract dynamic visual feature vectors (video tokens) that span from local to global contexts.
  - For text instructions, the JSON-formatted text is vectorized using a standard tokenizer and an embedding layer.
(2) Cross-Modal Feature Fusion: This framework adopts an additive fusion strategy. The feature vectors from both video and text are combined through vector addition (⊕) and then passed through a Fusion Layer for non-linear transformation. This process generates a unified multimodal input sequence for the language model decoder.
(3) LoRA-based decoder fine-tuning: This is the core of our PEFT methodology. During fine-tuning, the original weight matrices within the Qwen2.5 LM decoder (primarily in the multi-head attention and feed-forward network layers) are kept frozen. We employ the low-rank adaptation (LoRA) technique, which injects a trainable bypass path composed of two low-rank matrices, *A* and *B*, in parallel with each frozen matrix *W*. The model’s update, ΔW, is simulated by the product BA, such that the new output is $h^{\prime }=Wx+BAx$. For initialization, matrix *A* follows a Gaussian distribution $N(0,\sigma^{2})$, while matrix *B* is initialized to zero to ensure stability at the start of fine-tuning [26,27].
(4) Task-Specific Output Layer: The feature vectors output by the decoder are normalized by a Qwen2RMSNorm layer and then fed into a trainable Linear output head, which maps them to the final fault diagnosis results.

**Figure 4:**
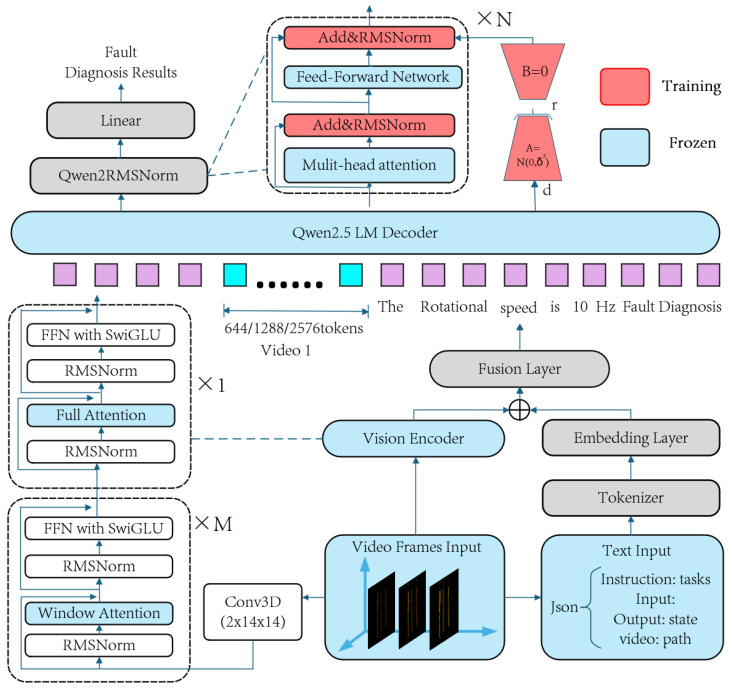
A PEFT framework integrating the Qwen2.5-VL model for video-based fault diagnosis.

The overall framework is illustrated in Figure 4.

In summary, by only fine-tuning the newly added LoRA adapters and the task-specific output head, this framework efficiently adapts the model to the specialized video diagnosis task while preserving its powerful pre-trained capabilities [28].

## 3. Results

### 3.1. Dataset Introduction

In this experiment, the relative position between the event camera and the rolling bearing was carefully designed to ensure data quality. The camera was positioned at a distance of 20 cm, measured perpendicularly from the top surface of the rolling bearing, which served as the zero reference plane. Concurrently, the camera’s viewing angle was set to 0°, aimed perpendicularly at the top of the bearing. To maintain consistent lighting conditions, an external artificial light source was applied.

Data were collected for three distinct bearing health conditions: normal, outer ring fault, and inner ring fault. Normal refers to a bearing in a healthy, undamaged state. An outer ring fault (OuterH) refers to a rectangular groove, approximately 4 mm long and 2 mm wide, artificially created on the outer raceway of the bearing through machining. This fault is designed to simulate localized defects such as spalling or pitting caused by fatigue in practical operating conditions. An inner ring fault (InnerH) involves a local defect of the same dimensions created on the inner raceway.

**Figure 5:**
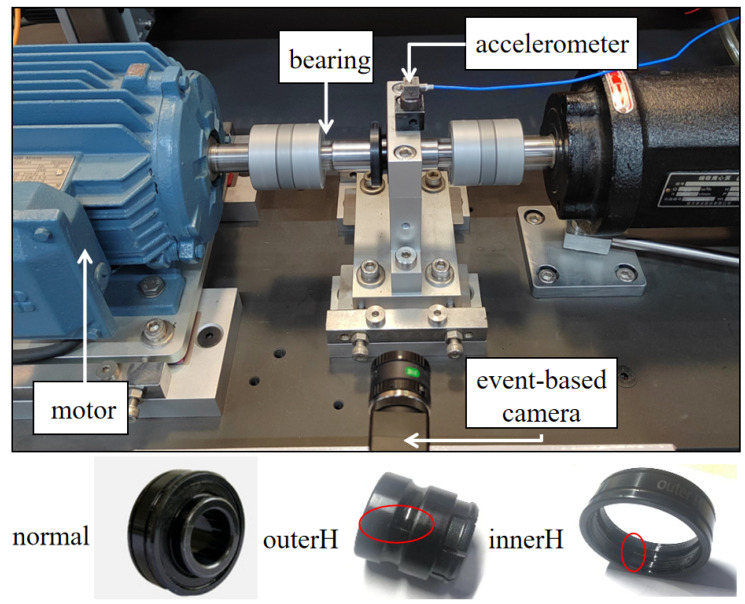
Photograph of the experimental motor and 3 types of bearing states.

To comprehensively evaluate the model’s generalization ability, all experiments were conducted under four different motor rotational speeds: 5 Hz, 8 Hz, 10 Hz, and 15 Hz. For each set of experimental conditions, the duration of each data recording was 30 s. To validate the effectiveness of the proposed fault diagnosis framework, we designed and conducted two distinct experiments. The data utilized in this study were sourced from the Fault Diagnosis Laboratory at the Institute of High-end Equipment, Xi’an Jiaotong University. The captured image and the three types of bearing states are presented in Figure 5.

**Figure 6:**
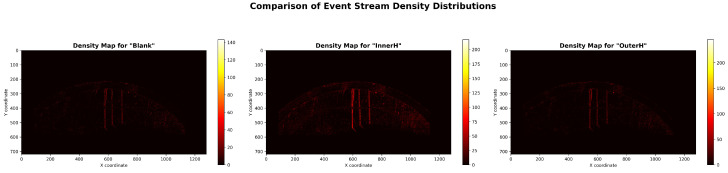
Density distribution maps of the original event streams under three conditions.

**Figure 7:**
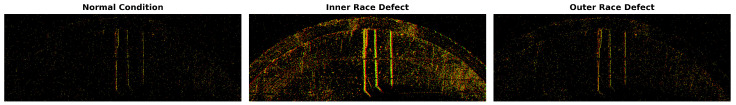
Images after time surface processing under three conditions.

This study uses three real-world motor fault datasets. They are referred to as Dataset A, Dataset B, and Dataset C. Each dataset was designed for different operating conditions and fault modes. Each dataset includes four rotational speeds and three damage types. Figure 6 shows the raw event stream density from the event camera. Figure 7 displays the processed images after applying the temporal surface method [29].

The selection of the time window, Δt, and the decay constant, τ, is critical. These parameters ensure effective processing and high-quality feature maps. We conducted exploratory experiments to find the optimal settings. Our selection process was guided by physical intuition. The time window Δt must be long enough to capture a complete fault impact cycle. However, it should not be so long that it causes signal aliasing. Similarly, the decay constant τ must balance two competing needs. It needs to preserve motion continuity while also highlighting transient impact details.

Our tests were based on the motor’s rotational periods at different speeds. For example, the period is 100 ms at 10 Hz and approximately 67 ms at 15 Hz. We tested three representative values for Δt: 20 ms, 50 ms, and 100 ms. The 20 ms value was for high-speed transient details. The 50 ms value was for cycles at medium-to-low speeds. The 100 ms value was for full cycles at low speeds. For each Δt, we tested three corresponding τ values. These were proportional to Δt, set as 0.2Δt, 0.5Δt, and 0.8Δt. This allowed us to evaluate how different decay rates affect feature clarity.

Our evaluation combined two methods. We used qualitative observation and quantitative assessment. For the qualitative part, we checked the clarity of fault textures in the generated images. For the quantitative part, we measured diagnostic accuracy on the validation set. The results showed one parameter set was optimal: Δt=50 ms and τ=25 ms. This combination produced the clearest and most consistent images of periodic impact patterns. It did not cause significant motion blur. It also achieved the highest preliminary classification performance.

Therefore, we selected the final parameter set of (Δt=50ms,τ=25ms). We applied these parameters to all subsequent experiments. This ensures the consistency and comparability of our results.

Different fault types exhibit significantly different visual signatures after temporal surface processing. These signatures are highly correlated with their respective physical vibration characteristics. A normal bearing, due to its smooth operation, typically presents as low-density, uniformly distributed, continuous stripes in the temporal surface image. This clearly reflects the regular, periodic motion of the rolling elements on the raceway, resulting in the lowest overall level of event activity. When an outer race fault is present, the location of the defect in space is fixed. Therefore, periodic, high-energy impact events are generated at the same spatial location each time a rolling element passes over it. This forms a series of high-intensity vertical stripes in the temporal surface image that are fixed in their horizontal position. The frequency of these stripes corresponds precisely to the ball pass frequency of the outer race. The pattern is clear and stable, and the overall event density is much higher than in the normal state. In stark contrast, the defect in an inner race fault rotates along with the inner race. This causes the location of the impact to change continuously with the shaft’s rotation. The impacts are typically most intense in the load-bearing zone. Consequently, its visual signature is a series of high-intensity impact stripes that exhibit a periodic positional modulation or a sweeping pattern in the horizontal direction. Although the impacts themselves are periodic and correspond to the ball pass frequency of the inner race, the movement of the defect widely distributes these high-energy events across the entire field of view. This makes the overall image appear more complex and cluttered than that of an outer race fault, and the event density is the highest. However, this is not random noise, but a recognizable, deterministic pattern caused by the rotational modulation [30,31].

These datasets were subsequently partitioned into training, validation, and testing sets. To comprehensively evaluate the model’s performance, we established two experimental schemes based on different input data representations. The detailed settings for each scheme are presented in Table 2.

### 3.2. Experimental Environment

The experiments were conducted in the following hardware and software environment. Hardware: Intel Xeon series multi-core CPU, Nvidia GeForce RTX 4090 GPU (24 GB VRAM). Software Environment: The system was running on Ubuntu 22.04, utilizing Python 3.11, the PyTorch 2.6 deep learning framework, and CUDA 12.4. VS Code was used as the primary development tool.

The model fine-tuning process is based on Alibaba’s open-source Qwen2.5VL-7B, a pre-trained large vision–language model (LVLM). This model supports both Chinese and English and has publicly available pre-trained weights, which facilitates subsequent fine-tuning on specialized tasks. We utilize LoRA low-rank adaptation techniques for lightweight fine-tuning of Qwen2.5VL-7B. This approach allows the model to effectively incorporate bearing fault feature knowledge while reducing VRAM usage and training complexity. During the model inference phase, the fault diagnosis task requires stable and consistent results. We therefore set the model’s generation temperature parameter to 0.01. This minimizes the randomness of the output and ensures reliable consistency for each diagnostic result.

### 3.3. Data Processing

**Figure 8:**
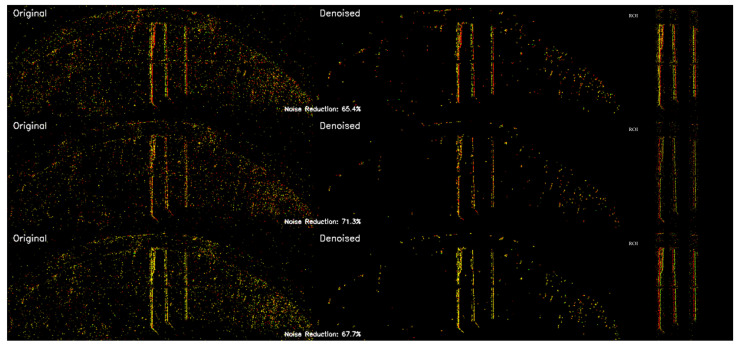
Comparison of bearings in three conditions after time surface processing, ROI cropping, and denoising.

To construct a corpus suitable for large model training, we preprocess the raw data acquired from the event camera. Following the processing framework, we use a sliding event window to extract sample segments. Each segment has a duration of 0.5 s with a window stride of 0.25 s. This ensures that fault information is maximally preserved during sequence segmentation while minimizing edge information loss. A total of 1548 raw samples were extracted from all the data. These samples undergo a series of preprocessing steps, including conversion to time surfaces, region of interest (ROI) cropping, and denoising, to generate complete event frame images. Finally, these event frames are concatenated to create trainable videos. The video parameters were chosen to balance diagnostic feature integrity with data availability. Based on our analysis of the bearing’s characteristic fault frequencies, a duration of 0.5 s was selected, as it reliably captures at least 2–3 complete fault cycles, which is crucial for robust diagnosis. A shorter duration, such as 0.25 s, would capture one cycle at most, potentially missing key diagnostic patterns, while a longer duration of 1 s would have drastically reduced the total number of available training samples. In parallel, based on our comparative experiments and the recommendations from the Qwen2.5-VL technical report, we determined that an equivalent frame rate of 30 fps (15 frames per 0.5 s sample) is optimal for the model’s video processing pipeline [32]. A schematic of this process is shown below in Figure 8.

For each sample, we then concatenate its corresponding rotational speed and fault class label into a descriptive sentence, which serves as the input corpus for the large model.

To validate the effectiveness of multimodal large model fine-tuning and to investigate the model’s ability to learn temporal information embedded in videos, this study designed two comparative experimental schemes. This approach ensures data source consistency between the two schemes [33,34]:

- Scheme 1 (Image-based Diagnosis): This scheme uses keyframes randomly extracted from the original videos via OpenCV as the model’s input.
- Scheme 2 (Video-based Diagnosis): This scheme directly uses the unmodified raw video sequences as input.

Subsequently, we construct the training, validation, and testing sets for model fine-tuning, partitioned in a 70%, 15%, and 15% ratio, respectively. During the partitioning process, we ensured a balanced distribution of samples for each fault type across the sets to mitigate any potential bias caused by class imbalance during model training. The detailed dataset split is presented in Table 3.

### 3.4. Hyperparameter Settings

We needed to accommodate the limited sample size of this study and prevent overfitting. Therefore, we specifically designed the configuration for parameter-efficient fine-tuning. The choice of LoRA hyperparameters was guided by established practices and preliminary experiments. This aimed to ensure sufficient model capacity while maintaining computational efficiency for this proof-of-concept study.

Specifically, for the LoRA parameters, we set the rank to $r_{\mathrm{rank},\mathrm{lora}}=8$. This value is a common choice that provides a good balance between parameter efficiency and expressive power. Our preliminary tests indicated that a higher rank (e.g., 16) yielded negligible performance gains at the cost of significantly increased computational overhead, while a lower rank could risk underfitting. The scaling factor was set to $\alpha_{\mathrm{alpha},\mathrm{lora}}=16$, following the standard convention of setting α=2×r. This ratio is widely adopted, as it helps stabilize the training process by appropriately scaling the low-rank updates [35].

For the training parameters, we employed a fine-grained learning rate of $\eta_{\mathrm{lr},\mathrm{model}}\;=\;1\times 10^{-5}$, a batch size of $b_{\mathrm{batch},\mathrm{model}}=2$, and a total of 4000 training iterations. Furthermore, the LoRA adapters were applied to multiple key layers within the model, including q_proj and v_proj, to enable comprehensive fine-tuning of the model’s behavior. Other detailed parameters are listed in the aforementioned Table 4.

### 3.5. Evaluation of Diagnostic Accuracy Under Various Operating Conditions

After determining the main hyperparameters, we conducted fault diagnosis experiments on a single dataset for the two experimental schemes designed in this study: Scheme 1 and Scheme 2. During the experimental process, the model was fine-tuned separately using data from each modality. After each training epoch, the model’s performance was evaluated on the validation set, and the best-performing weights were saved for final testing. We conducted independent training for both schemes and evaluated the fault classification accuracy on their respective test sets.

**Figure 9:**
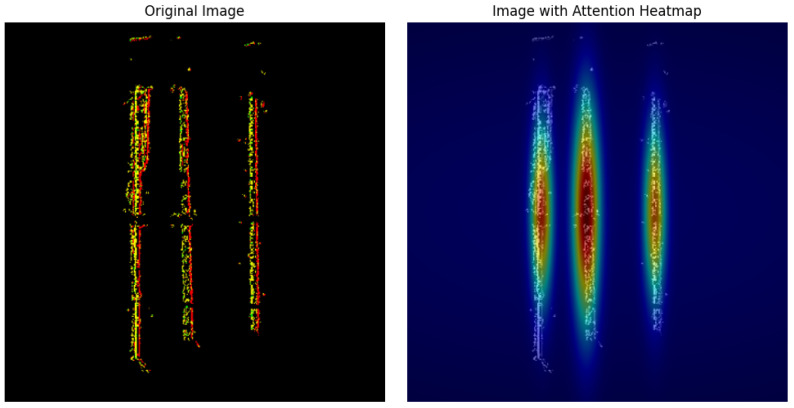
Attention weight heatmap of the last visual transformer layer.

As shown in Table 5, the proposed fault diagnosis method based on a large model achieved excellent performance under both schemes. To further investigate the model’s internal decision-making mechanism, we performed a visualization of its attention weights. Figure 9 displays the attention heatmap from the final visual transformer layer of the model when processing video input. It can be clearly seen from the figure that the model’s attention is highly concentrated on vertically elongated, strip-like regions that extend along the time dimension. This indicates that the model successfully captures the continuous motion patterns and fault features generated by the bearing’s rotation over time, rather than merely focusing on isolated feature points in a static image. This precise focus on the dynamic process is key to the model’s ability to achieve high diagnostic accuracy [36].

**Figure 10:**
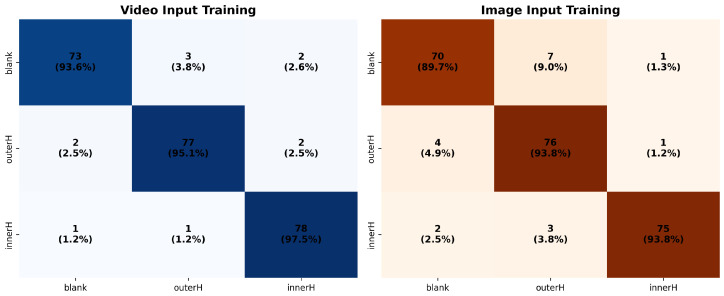
Confusion matrices for Scheme 1 (event frame-based) and Scheme 2 (video-based).

The confusion matrices for both schemes are presented in Figure 10.

- Scheme 1 (Event Frame-based Diagnosis): The overall diagnostic accuracy on the test set is 92.47%. Specifically, the per-class identification accuracies for the normal state, outer race fault (outerH), and inner race fault (innerH) are 89.74%, 93.83%, and 93.75%, respectively.
- Scheme 2 (Video-based Diagnosis): The overall diagnostic accuracy reaches 95.44%, showing a significant improvement over Scheme 1. Its per-class identification accuracies for the normal, outer race fault, and inner race fault categories are 93.59%, 95.18%, and 97.50%, respectively, demonstrating a comprehensive performance advantage across all classes.

The experimental results clearly indicate that Scheme 2, which directly utilizes raw video as input, outperforms Scheme 1. This is true for both overall performance and per-class identification accuracy. This strongly validates that by processing the video sequence directly, the model effectively learns the embedded temporal information. It thereby constructs more discriminative fault features. Scheme 1 achieved a respectable level of accuracy. However, its relatively lower performance on the normal state (89.74%) may suggest that the static representation from event frames is sometimes insufficient. It cannot always fully distinguish it from certain fault modes. In contrast, Scheme 2 achieved an accuracy of over 93% for all classes, demonstrating stronger robustness and generalization capability.

**Figure 11:**
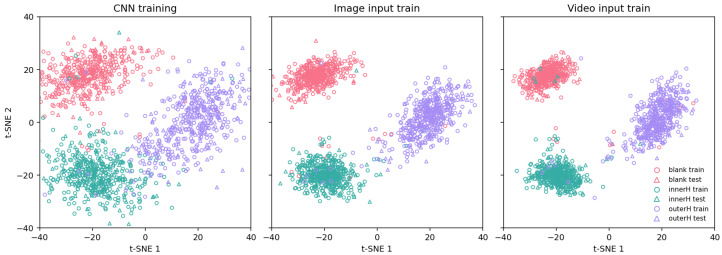
t-SNE visualization for different model inputs: baseline CNN, event frames, and video.

To visually validate our quantitative results, we used the t-SNE technique [37]. This method helps visualize the feature distribution from the model’s final layer. High-dimensional feature vectors are projected into a two-dimensional space for this analysis. We compared the visualization results for three inputs: a baseline CNN, event frames (Scheme 1), and video (Scheme 2) [38]. The results are presented in Figure 11.

- For the baseline CNN model: The t-SNE plot shows that although the feature vectors form three general clusters, there is significant overlap between the different classes. In particular, the feature distributions for the outer race fault (outerH) and inner race fault (InnerH) states are quite close. This indicates that the discriminative power of static spatial features extracted solely by a basic CNN is relatively limited, making it difficult to delineate clear decision boundaries.
- For Scheme 1 (Event Frame-based): The t-SNE plot similarly forms three discernible clusters, showing a significant improvement in feature separability compared to the baseline CNN model. As seen in the plot, the inter-cluster overlap is substantially reduced, with the majority of samples correctly aggregated within their respective class clusters. This demonstrates that the sparse spatial information captured by the event camera, which embodies motion changes, provides the model with more discriminative features than static images. Nevertheless, despite the good overall separation, a few instances of sample confusion can still be observed at the cluster boundaries, indicating that while its feature discrimination is strong, it has not yet reached an ideal state of complete separation.
- For Scheme 2 (Video-based): The t-SNE visualization reveals three highly separable clusters. The large inter-cluster distances and high intra-cluster compactness are highly consistent with the quantitative accuracy of 95.44%, robustly demonstrating that the dynamic spatiotemporal features learned from the complete video sequence possess the most powerful discriminative ability.

In summary, the t-SNE visualization results are consistent with the conclusions from the quantitative analysis. The comparison shows a progressive enhancement in the model’s feature extraction and discrimination capabilities, moving from static images (CNN), to sparse dynamic information (event frames), and finally to dense spatiotemporal information (video). Specifically, the video input, containing complete temporal information, enables the model to construct a feature space with the clearest decision boundaries and the strongest discriminative power, thereby achieving the most stable and reliable fault diagnosis.

### 3.6. End-to-End Processing Latency Analysis

To evaluate the feasibility of the proposed method in industrial applications, we conducted a comprehensive end-to-end processing latency analysis. The total delay was broken down into key stages from initial data acquisition to the final diagnostic result, with all tests performed on an Nvidia GeForce RTX 4090.

The analysis revealed that the event acquisition stage is a hardware-level response with negligible time cost (<1 ms). The data preprocessing stage, which includes denoising the raw event stream, ROI cropping, generating 15 time-surface frames, and encoding them into an MP4 video sequence, took approximately 215 ms on average. The model inference stage, where the Qwen2.5-VL-7B model processes the video to generate the final diagnostic result, was the most time-consuming, taking approximately 2530 ms.

Consequently, the total end-to-end latency of the system is approximately 2746 ms, with over 92% of this time consumed by the model inference stage. For a motor operating at 3000 rpm (50 Hz), a single rotation completes in just 20 ms. Our system’s current processing time of approximately 2750 ms significantly exceeds this real-time requirement, indicating that the current method does not meet the demands for immediate-response real-time control or equipment protection systems. However, its performance is well suited for applications such as periodic condition monitoring, offline data analysis, and predictive maintenance planning [18].

## 4. Conclusions

The core contribution of this study is the pioneering proposal and validation of a novel fault diagnosis paradigm based on fine-tuning an event camera with the Qwen2.5-VL multimodal large model. We innovatively used the dynamic event stream from an event camera as the model’s input, successfully combining the high temporal resolution of non-contact sensing with end-to-end learning. Experimental results strongly demonstrate the paradigm’s effectiveness. The model exhibited excellent diagnostic performance, achieving 92.47% accuracy on static images and 95.44% on dynamic videos. This confirms that event camera data serves as a high-information-density source that large models can effectively interpret.

Despite these promising results, a full evaluation of the technology’s feasibility and challenges is necessary before industrial deployment. Technology and cost are primary obstacles. The Qwen2.5-VL-7B model’s high GPU requirements of over 24 GB and slow inference speed of approximately 2–3 s per diagnosis limit its real-time deployment on edge devices. While the initial investment for an event camera is higher than for traditional sensors, its long-term cost effectiveness is competitive because a single camera can monitor multiple components, and its non-contact nature significantly reduces installation and maintenance costs.

More importantly, the robustness and feasibility of event cameras strongly support their industrial application. They are suitable for harsh conditions due to a wide operating temperature range from −40 °C to 85 °C, inherent immunity to electromagnetic interference, and a high protection rating of up to IP65. Furthermore, standard hardware interfaces and network capabilities facilitate easy integration and scalability, enabling remote and large-scale monitoring.

Nevertheless, challenges in operation and generalization remain. The method’s effectiveness depends on optimal camera placement, and its ability to generalize across different devices and operating conditions requires further validation.

Future work will directly address these challenges. We will focus on researching model compression techniques and conducting performance tests on embedded platforms like the Jetson Orin to achieve lightweight, real-time edge inference. Concurrently, we plan to pursue a deep fusion of event camera data with traditional sensor information like temperature and sound to build a more robust and comprehensive diagnostic system. We will also validate its generalization on more diverse industrial datasets [39].

In conclusion, this study provides a revolutionary non-contact, end-to-end, and high-precision solution for industrial equipment health monitoring. It not only validates the feasibility of this technological path but also reveals its immense potential and broad prospects for advancing industrial intelligence.

## Figures and Tables

**Table 1:** Field composition and content definition of multimodal fault diagnosis samples.

Field	Data Type	Content Definition
instruction	Text	Natural language instruction for the task.
input	Text	Additional context for the query (unused).
output	Text	Expected answer (bearing health status).
videos	Video Data	MP4 video from event data.

**Table 2:** Experimental settings and validated capabilities.

Diagnosis Scheme	Data Type	Train/Val/Test Samples	Diagnosis Capability
Event Frame-based	Image	1077 / 232 / 239	Basic diagnosis
Video-based	Video	1077 / 232 / 239	Diagnosis with time series

**Table 3:** Details of the dataset split.

Dataset Split	Fault Class	Videos
Training Set	normal	352
	innerH	360
	outerH	365
	Subtotal	1077
Validation Set	Normal	76
	innerH	77
	outerH	79
	Subtotal	232
Test Set	Normal	78
	innerH	80
	outerH	81
	Subtotal	239
Total	—	1548

**Table 4:** Hyperparameter settings for the model and fine-tuning process.

Parameter Type	Parameter	Value	Description
Language Module	$l_{\mathrm{cutoff},\mathrm{llm}}$	1024	Max input length for the language module
Adapter Parameters	$n_{\mathrm{neural},\mathrm{ifdm}}$	2048	Number of neurons in the adapter
LoRA Parameters	$r_{\mathrm{rank},\mathrm{lora}}$	8	The rank of the LoRA low-rank matrices
	$\alpha_{\mathrm{alpha},\mathrm{lora}}$	16	The learning rate scaling factor for LoRA
	$\delta_{\mathrm{dropout},\mathrm{lora}}$	0.1	Dropout rate for the LoRA layers
Training Parameters	$\eta_{\mathrm{lr},\mathrm{model}}$	$1\times 10^{-5}$	Learning rate
	$b_{\mathrm{batch},\mathrm{model}}$	2	Batch size.
	$w_{\mathrm{steps},\mathrm{model}}$	10	Warmup steps for the learning rate scheduler
	$d_{\mathrm{decay},\mathrm{model}}$	0.01	Weight decay.
	$g_{\mathrm{grad},\mathrm{model}}$	1	Gradient accumulation steps
	$\alpha_{\mathrm{alpha},\mathrm{model}}$	1	Loss weight parameter
	$\beta_{\mathrm{beta},\mathrm{model}}$	1	Loss weight parameter
	$n_{\mathrm{iter},\mathrm{model}}$	4000	Number of training iterations

**Table 5:** Training progression: core performance metrics comparison across epochs.

Training Scheme	Epoch	Acc.	W. Prec.	W. Recall	W. F1	Kappa
Image Input	**1**	85.21%	85.35%	85.21%	85.26%	77.82%
	**2**	88.34%	88.47%	88.34%	88.39%	82.51%
	**3**	90.89%	91.02%	90.89%	90.94%	86.34%
	**4**	92.47%	92.61%	92.47%	92.50%	88.70%
Video Input	**1**	87.45%	87.58%	87.45%	87.49%	81.18%
	**2**	91.56%	91.68%	91.56%	91.60%	87.34%
	**3**	93.87%	93.95%	93.87%	93.90%	90.81%
	**4**	95.40%	95.41%	95.40%	95.39%	93.09%

Acc. = Accuracy, W. Prec. = Weighted Precision, W. Recall = Weighted Recall, W. F1 = Weighted F1-Score.

## Data Availability

The data presented in this study are available upon request from the corresponding author.
